# Mutational profile and genotype/phenotype correlation of non-familial pheochromocytoma and paraganglioma

**DOI:** 10.18632/oncotarget.27194

**Published:** 2019-10-15

**Authors:** Shatha Albattal, Meshael Alswailem, Yosra Moria, Hindi Al-Hindi, Majed Dasouki, Mohamed Abouelhoda, Hala Aba Alkhail, Entissar Alsuhaibani, Ali S. Alzahrani

**Affiliations:** ^1^ Department of Molecular Oncology, King Faisal Specialist Hospital and Research Centre, Riyadh 11211, Saudi Arabia; ^2^ Department of Medicine, King Faisal Specialist Hospital and Research Centre, Riyadh 11211, Saudi Arabia; ^3^ Department of Pathology and Laboratory Medicine, King Faisal Specialist Hospital and Research Centre, Riyadh 11211, Saudi Arabia; ^4^ Department of Genetics, King Faisal Specialist Hospital and Research Centre, Riyadh 11211, Saudi Arabia; ^5^ Saudi Human Genome Program, King Abdulaziz City for Science and Technology, Riyadh 11211, Saudi Arabia; ^6^ Faculty of Science, King Saud University, Riyadh 11211, Saudi Arabia

**Keywords:** pheochromocytoma, paraganglioma, mutations, NGS, SDHB

## Abstract

About 30%–40% of patients with pheochromocytoma (PCC) and paraganglioma (PGL) have underlying germline mutations in certain susceptibility genes despite absent family history of these tumors. Here, we present mutational profile of 101 such patients with PCC/PGL (PPGL) from the highly consanguineous population of Saudi Arabia.

**Results:** Of 101 cases with PPGL, 37/101 (36.6%) had germline mutations. Mutations were detected in 30 cases by PCR and direct Sanger sequencing and in 7 additional cases by NGS. The most commonly mutated gene was *SDHB* (21/101 cases, 20.8%) and the most common *SDHB* mutation was c.268C>T, p.R90X occurring in 12/21 (57%) cases. Mutations also occurred in *SDHC* (4/101, 3.96%), *SDHD* (3/101, 3%), *VHL* (2/101, 2%) and *MAX* (2/101, 2%) genes. The following genes were mutated in 1 patient each (1%), *RET, SDHA, SDHAF2, TMEM127* and *NF1*. Metastatic PPGL occurred in 6/21 cases (28.6%) with *SDHB* mutations and in 1 case with *SDHAF2* mutation.

**Patients and Methods:** DNA was isolated from peripheral blood (53 patients) or from non-tumorous formalin fixed paraffin embedded (FFPE) tissue (48 patients). PCR and direct Sanger sequencing of *RET, SDHx, VHL, MAX* and *TMEM127* genes were performed. Cases without mutations were subjected to whole exome sequencing using next generation sequencing (NGS).

**Conclusion:** About 37% of PPGL without family history of such tumors harbor germline mutations. The most commonly mutated gene is *SDHB* followed by *SDHC*, *SDHD, VHL, MAX* and rarely *RET, SDHA, SDHAF2, TMEM127* and *NF1*. *SDHB* mutations were associated with metastatic PPGL in more than a quarter of cases.

## INTRODUCTION

Pheochromocytoma and paraganglioma (PPGL) are chromaffin cell-derived neuroendocrine tumors [1]. While having been considered mostly sporadic for long time, it has become clear over the last 2 decades that 30–40% of these tumors are due to underlying germline mutations in one of several susceptibility genes [2–4]. Nearly 30 genes have been identified with germline or somatic mutations in PPGL [3]. Apart from the well-known genes such as *RET, NF1, VHL, SDHx, TMEM127, and MAX*, many of the new genes have been identified in the last 7 years that include *HIF2A-EPAS1, FH, H-RAS, PHD1, MDH2, ATRX, H3F3A, CSDE1, MAML3* and *IRP1* (summarized in reference [4]). These genes involve at least 3 signal transduction pathways, the pseudohypoxemia, the tyrosine kinase, and the WNT pathways [3, 5]. Much more recently discovered predisposing genes for PPGL include *DLST* [6], *SLC25A11* [7] *and DNMT3A* [8]. The discovery of new genes has been propagated by major advances in technology. Next Generation Sequencing (NGS) technology has made sequencing of the whole exome fairly routinely available. Many studies have been published on the molecular genetics of PPGL using this technology [3–5, 9–12]. These studies have shown some ethnic differences in the rates of mutations and the underlying genetic landscape [12–15]. There have not been comprehensive studies from the Middle East region and none from the Arabic population. These populations are homogeneous with high rates of consanguinity making them ideal for studying hereditary diseases [16]. In this paper, we report the underlying genetic mutations and the genotype/phenotype correlation in a large series of patients with PPGL from the highly consanguineous population of Saudi Arabia.

## RESULTS

### Patients' characterstics

A total of 101 patients were included in this study and their clinical and pathological characteristics are summarized in Table 1.

**Table 1:** Age, sex and pathological features of 101 cases of PPGL

Characteristic	Number or Frequency
Age (yrs) median (Range)	38 (8–81)
Sex F: M	61:40
Tumor size (cm), Median (Range)	5 (1–24)
Vascular Invasion	10 (9.9%)
Capsular invasion	19 (18.8%)
Distant Metastasis	10 (9.9%)
**Sites**	
PCC (4 Bilateral)	32 (31.7%)
Abdominal PGL	26 (25.7%)
Head/Neck PGL (2 bilateral)	39 (38.6%)
Other sites	2 (1.99%)
Multiple sites (including 4 bilateral PCC and 2 bilateral head/neck PGL)	8 (7.9%)

### Mutational analysis and genotype/phenotype correlation

**Figure 1:**
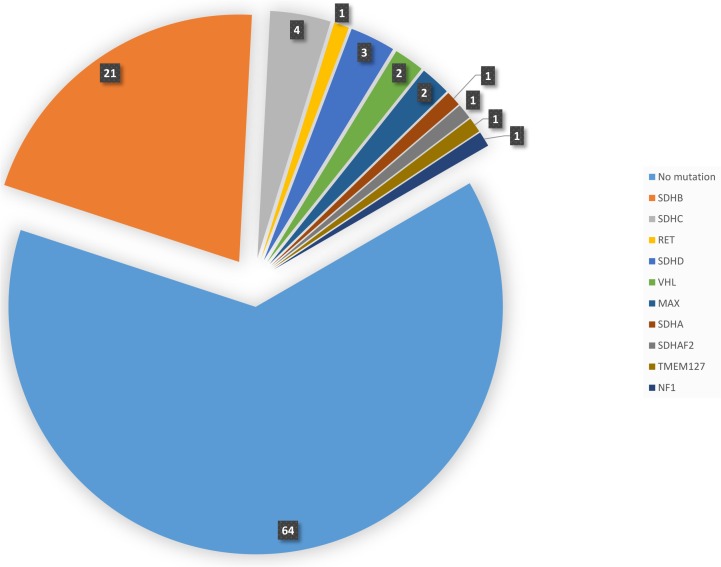
Pie diagram showing the distribution and number of cases with germline mutations in different gene.

Germline mutations were detected in 30 cases by PCR and direct Sanger sequencing. The remaining cases were subjected to whole exome sequencing using NGS as direct Sanger sequencing either did not reveal mutations in the known genes that were tested or was unsuccessful. NGS identified additional 7 cases with pathogenic variants. These 7 cases with NGS-detected mutations were subsequently confirmed by Sanger sequencing. Overall, of 101 sporadic cases of PPGL, 37 (36.6%) had germline mutations while 64 patients (63.4%) had no mutations in any of the genes known to be involved in the pathogenesis of PPGL (Table 2 and Figure 1). The most commonly mutated gene was *SDHB* (21/101 cases, 20.8%) followed by *SDHC* (4/101, 3.9%). *SDHD, VHL* and *MAX* genes were mutated in 3 (3%), 2 (2%) and 2 (2%) cases, respectively. The following genes were mutated in 1 patient each (1%), *RET*, *SDHA, SDHAF2, TMEM127* and *NF1*.

**Table 2:** Overall results of genomic profiling of 101 cases of apparently sporadic PPGL

Diagnosis	Total	Mutation-positive	*SDHB*	*SDHD*	*SDHC*	*SDHA*	*SDHAF2*	*RET*	*VHL*	*NF1*	*MAX*	*TMEM127*
Unilateral PCC (1 metastatic)	28	4	1				1		1	1		
Bilateral PCC	4	3							1		2	
Abd. PGL (6 metastatic)	26	16	12		2			1				1
H/N PGL (2 metastatic)	37	10	5	2	2	1						
Bilateral H/N PGL	2	2	1	1								
Abd. and H/N PGL	1	1	1									
PCC, abd. and H/N PGL	1	1	1									
Bladder PGL	1	0										
Intradural PGL	1	0										
**Total**	**101**	**37 (36.6%)**	**21 (20.8%)**	**3 (3%)**	**4 (3.9%)**	**1 (1%)**	**1 (1%)**	**1 (1%)**	**2 (2%)**	**1 (1%)**	**2 (2%)**	**1 (1%)**

### *SDHB* mutations

The majority of cases with *SDHB* mutations presented with abdominal PGL (14/21 cases, 66.7%) (Table 3) including one patient with combined abdominal and head and neck PGL. Five cases presented with head/neck PGL of whom one case had bilateral carotid body tumors associated with the c.689G>A, p.R230H mutation. Two cases presented with PCC (one isolated and one with mediastinal and head and neck PGL). Six cases (28.6%) developed distant metastasis (Table 3). *SDHB* is the most commonly mutated gene in our series (21/37 mutations, 56.8%). The non-sense mutation c.268C>T, p.R90X was the most frequent mutation occurring in 12 out of 21 cases (57%) with *SDHB* mutations. In 4 cases (33%) with this mutation, the disease was metastatic (Table 3).

**Table 3:** *SDHB* mutations (NM_003000.2) in 21 cases of PPGL

Diagnosis	Metastatic	Tissue	Sequencing	Mutation *Nucleotide change*	Mutation *Amino acid change*	Variant status
Abdominal PGL	Yes	Blood	Sanger	c.412G>A	p.D138N	Known Pathogenic [51]
Abdominal PGL	Yes	Blood	Sanger	c.689G>A	p.R230H	Known Pathogenic [52]
Abdominal PGL	Yes	Blood	Sanger	c.268C>T	p.R90X	Known Pathogenic [53]
Abdominal PGL	Yes	Blood	Sanger	c.268C>T	p.R90X	Known Pathogenic [53]
Abdominal PGL	Yes	Blood	Sanger	c.268C>T	p.R90X	Known Pathogenic [53]
Abdominal PGL	No	FFPE	Sanger	c.343C>T	p.R115X	Known Pathogenic [54]
Abdominal PGL	No	Blood	Sanger	c.268C>T	p.R90X	Known Pathogenic [53]
Abdominal PGL	No	FFPE	Sanger	c.268C>T	p.R90X	Known Pathogenic [53]
Abdominal PGL	No	FFPE	Sanger	c.268C>T	p.R90X	Known Pathogenic [53]
Abdominal PGL	No	FFPE	Sanger	c.268C>T	p.R90X	Known Pathogenic [53]
Abdominal PGL	No	Blood	Sanger	c.268C>T	p.R90X	Known Pathogenic [53]
Abdominal PGL	No	FFPE	Sanger	c.268C>T	p.R90X	Known Pathogenic [53]
Abdominal PGL	No	FFPE	NGS	c.637dupA	p.M213fs	Novel, likely pathogenic
H/N PGL	No	Blood	Sanger	c.689G>A	p.R230H	Known Pathogenic [55]
H/N PGL	No	Blood	Sanger	c.409A>G	p.K137E	Novel, likely pathogenic
H/N PGL	No	Blood	Sanger	c.268C>T	p.R90X	Known Pathogenic [53]
H/N PGL	No	FFPE	Sanger	c.79C>T	p.R27X	Known Pathogenic [2]
Bilateral H/N PGL	No	Blood	Sanger	c.689G>A	p.R230H	Known pathogenic [55]
Abdominal and H/N PGL	Yes	FFPE	Sanger	c.268C>T	p.R90X	Known Pathogenic [53]
Unilateral PCC	No	Blood	Sanger	c.268C>T	p.R90X	Known Pathogenic [53]
PCC, mediastinal PGL, H/N PGL	No	Blood	Sanger	c.689G>A	p.R230H	Known Pathogenic [54]

H/N, Head and Neck; PGL, Paraganglioma; PCC, Pheochromocytoma; FFPE, Formalin Fixed Paraffin Embedded tissue

### *SDHC* mutations

Four patients had *SDHC* mutations. These mutations were missense in 3 cases and a splice site mutation in one case (Tables 2 and 4). Two of these 4 cases had abdominal PGL while the other two had head and neck PGL. All of these were benign tumors.

### *SDHD* mutations

The most common variant found was c.34G>A, p. G12S occurring in 7/10 cases with *SDHD* variants (70%). However, the role of this variant in the pathogenesis of PPGL has been controversial. Excluding this variant, another 3 cases had germline *SDHD* mutations (Table 4). They all presented with head and neck PGL (bilateral in one case). Interestingly, a case with the nonsense mutation c.15G>A, p.W5X presented with multiple recurrent head and neck PGLs (Table 4).

**Table 4:** Mutations in *RET*, *VHL*, *NF1*, *SDHA*, *SDHC*, *SDHD*, *SDHAF2*, *TEMEM127* and *MAX*

Diagnosis	Gene	Tissue	Sequencing	*Nucleotide change*	*Amino Acid Change*	Variant status
Abdominal PGL	RET	FFPE	Sanger	c.1900T>C	p.C634R	Known Pathogenic [56]
Unilateral PCC	VHL	Blood	Sanger	c.482G>A	p.R161Q	Known Pathogenic [2, 57]
Bilateral PCC	VHL	Blood	Sanger	c.355T>C	p.F119L	Known Pathogenic [58]
Unilateral PCC	NF1	Blood	NGS	c.4150G>A	p.E1384K	Novel, VUS
Unilateral H/N PGL	SDHA	Blood	Sanger	c.994C>T	p.P332S	Novel, likely pathogenic
Abdominal PGL	SDHC	FFPE	Sanger	c.305T>C	p.L102P	Novel, likely pathogenic
Abdominal PGL	SDHC	FFPE	Sanger	c.329C>T	p.P110L	Novel, likely pathogenic
H/N PGL	SDHC	Blood	NGS	c.78-2A>T	Splice site mutation	Known Pathogenic [59, 60]
H/N PGL	SDHC	Blood	Sanger	c.164A>G	p.H55R	Novel, likely pathogenic
H/N PGL	SDHD	FFPE	Sanger	c.184G>A	p.A62T	Novel, likely pathogenic
H/N PGL	SDHD	FFPE	Sanger	c.335C>T	p.T112I	Novel, likely pathogenic
Bilateral H/N PGL	SDHD	Blood	Sanger	c.15G>A	p.W5X	Likely pathogenic [2, 61]
Metastatic PCC	SDHAF2	Blood	NGS	c.438C>A	p.N146K	Novel, VUS
Abdominal PGL	TMEM127	Blood	NGS	c.281G>A	p.R94Q	Novel, likely pathogenic
Bilateral PCC	MAX	Blood	NGS	c.196C>T	p.R66X	Novel, likely pathogenic
Bilateral PCC	MAX	Blood	NGS	c.161T>A	p.I54N	Novel, likely pathogenic

VUS, Variant of Unknown significance; NGS, next generation sequencing

### *RET* mutations

Only one patient without family or personal history of multiple endocrine neoplasia type 2 presented with abdominal PGL and his genetic testing revealed the well-known c.1900T>C, p.C634R (Table 4). In addition, the c.2071G>A, p.G691S variant is a single nucleotide polymorphism (SNP) that was quite common occurring in 6 cases (5 PCC and 1 abdominal PGL).

### Other genes

A number of other known genes were mutated and presented with interesting manifestations (Table 4). *VHL* mutations were detected in 2 cases (Table 4). These cases presented with bilateral PCC in one case and unilateral PCC in another case (Table 4). *MAX* gene was mutated in 2 cases both of whom presented with bilateral PCC. A novel *SDHAF2* variant was found in one case of metastatic PCC. By in Silico analysis (Mutation taster and Polyphen2), this variant is disease causing and probably damaging with a score of 0.999. Other genes included *RET*, *TMEM127*, *SDHA* and *NF1*. Each was mutated once. The tumor type, location and the mutations in these genes are detailed in Table 4. The patient with *NF1* variant does not have features of neurofibromatosis. However, the variant was confirmed on Sanger sequencing and the in Silico analysis predicted it to be highly damaging.

#### Mutations by tumor type

##### Mutations in PCC

Our study included a total of 32 cases of PCC. These include 26 cases with unilateral, 4 with bilateral and 2 metastatic PCC. Twenty-four cases (75%) had no detectable germline mutations. The other 8 (8/32, 25%) cases harbored underlying germline mutations. The underlying genes and mutations are detailed in Table 5. Bilateral PCC occurred in 4 cases and the underlying genes were *MAX* in 2 cases, *VHL* in 1 case and no identifiable mutation in another case. In one unusual case with metastatic PCC, the underlying mutation was a novel *SDHAF2* variant that is likely pathogenic (Tables 4 and 5).

**Table 5:** Germline mutations in patients with PCC, abdominal PGL and head and neck PGL

Category	Diagnosis	Gene	Mutation (*Nucleotide change*)	*Mutation (Amino Acid Change)*
**Pheochromocytoma**	Unilateral PCC	*SDHB*	c.268C>T	p.R90X
	Unilateral PCC	*NF1*	c.4150G>A	p.E1384K
	Metastatic PCC	*SDHAF2*	c.438C>A	p.N146K
	Unilateral PCC	*VHL*	c.482G>A	p.R161Q
	Bilateral PCC	*VHL*	c.355T>C	p.F119L
	Bilateral PCC	*MAX*	c.196C>T	p.R66X
	Bilateral PCC	*MAX*	c.161T>A	p.I54N
	Unilateral PCC, Mediastinal PGL, H/N PGL	*SDHB*	c.689G>A	p.R230H
**Abdominal Paraganglioma**	Abdominal PGL	*RET*	c.1900T>C	p.C634R
	Abdominal PGL	*SDHB*	c.637dupA	p.M213fs
	Abdominal PGL	*SDHB*	c.268C>T	p.R90X
	Abdominal PGL	*SDHB*	c.268C>T	p.R90X
	Abdominal PGL	*SDHB*	c.343C>T	p.R115X
	Abdominal PGL	*SDHB*	c.268C>T	p.R90X
	Abdominal PGL	*SDHB*	c.268C>T	p.R90X
	Abdominal PGL	*SDHB*	c.268C>T	p.R90X
	Abdominal PGL	*SDHB*	c.268C>T	p.R90X
	Abdominal PGL	*SDHC*	c.305T>C	p.L102P
	Abdominal PGL	*SDHC*	c.329C>T	p.P110L
	Abdominal PGL	*TMEM127*	c.281G>A	p.R94Q
	Metastatic Abdominal PGL	*SDHB*	c.412G>A	p.D138N
	Metastatic Abdominal PGL	*SDHB*	c.689 G>A	p.R230H
	Metastatic abdominal PGL	*SDHB*	c.268C>T	p.R90X
	Metastatic Abdominal PGL	*SDHB*	c.268C>T	p.R90X
	Metastatic Abdominal PGL	*SDHB*	c.268C>T	p.R90X
	Metastatic abdominal and H/N PGL	*SDHB*	c.268C>T	p.R90X
**Head and neck paraganglioma**	Unilateral H/N PGL	*SDHA*	c.994C>T	p.P332S
	Unilateral H/N PGL	*SDHB*	c.409A>G	p.K137E
	Unilateral H/N PGL	*SDHB*	c.268C>T	p.R90X
	Unilateral H/N PGL	*SDHB*	c.79C>T	p.R27X
	Unilateral H/N PGL	*SDHB*	c.689G>A	p.R230H
	Unilateral H/N PGL	*SDHC*	c.78-2A>T	Splice site
	Unilateral H/N PGL	*SDHC*	c.164A>G	p.H55R
	Unilateral H/N PGL	*SDHD*	c.184G>A	p.A62T
	Unilateral H/N PGL	*SDHD*	c.335C>T	p.T112I
	Bilateral H/N PGL	*SDHB*	c.689G>A	p.R230H
	Bilateral H/N PGL	*SDHD*	c.15G>A	p.W5X

##### Mutations in PGL

Overall, 69 cases of PGL were included, of which 26 abdominal PGL (6 metastatic), 35 head and neck PGL, 1 combined abdominal and head/neck PGL, 1 combined PCC, abdominal and head/neck PGLs, 1 bladder PGL, 2 bilateral head/neck PGL, 2 metastatic head and neck PGL and 1 intradural spinal PGL (Table 2). The spinal PGL occurred in a 28-year old man who presented with a 14-month history of lower back pain radiating to both hips and difficulty in walking. He had no hyperadrenergic symptoms. MRI of the spine showed an intradural tumor of 1.4 cm size at lumbar spine 3 (L3) compressing the cauda equina. Surgical resection and histolpathological examination confirmed a diagnosis of PGL with typical microscopic picture and positive Chromogranin A stain. Germline mutations were found in 29 of 69 (42%) of these PGLs while 40 cases were negative for any underlying mutation. These cases with positive mutations include 17 cases of abdominal PGL (5 metastatic), 11 cases of head and neck PGL (two bilateral) and one case of abdominal and head and neck PGLs (Table 5). The details of these mutations are summarized in Table 5.

##### Multiple PPGL

Four patients had bilateral PCC (2 with *MAX*, 1 *VHL* and 1 without identifiable mutation) (Table 5), one patient had synchronous abdominal and head/neck PGL (*SDHB* R90X mutation) (Table 5), two had bilateral head and neck PGLs (one *SDHB* and 1 *SDHD* mutation) (Table 5), and 1 patient had metachronous PCC, abdominal PGL and head and neck PGL (p.R230H *SDHB* mutation) (Table 5).

##### Metastatic PCC and PGL

Overall, 10/101 patients (10%) had metastatic PPGL and 7 of them (70%) had underlying germline mutations. Two patients had metastatic PCC (one had no mutation and one had an *SDHAF2* mutation) (Tables 4 and 5). Six patients had abdominal PGL and *SDHB* mutations; one of them had both abdominal and head and neck PGLs (Table 3) and 2 patients had metastatic head and neck PGL (both negative for mutations). The first patient with head and neck PGL was a 38-year old lady who presented with a 7-cm left carotid body tumor that could not be completely resected as it was invading the surrounding structures and the carotid artery. Histopathologcal examination of the resected tumor confirmed the diagnosis of PGL with positive chromogranin A and synaptophysin stains and a Ki67 proliferation index of 3%. There was clear capsular and vascular invasion and positive margin. CT scan of the chest, abdomen and pelvis and MIBG whole body scan showed MIBG-avid bilateral lung metastases up to 1 cm in size. The patient was treated with surgery twice, chemotherapy and 3 doses of MIBG (cumulative dose 364 mCi) but continued to have progression of the lung metastases. She is currently stable with persistent bilateral lung metastases. The second patient was a 48-year old man who presented with a 4-year history of gradually enlarging right mid and upper neck mass with dizziness, headache and pain. CT scan of the neck, chest abdomen and pelvis showed a 6 × 4 cm right carotid body tumor and innumerable bilateral 1-1.5 cm lung, liver and skeletal metastases. These lesions were only faintly positive on MIBG but vey avid on octreotide whole body scan. Histopathological examination of the resected right carotid body tumor and biopsy from a liver lesion confirmed the diagnosis of metastatic carotid body tumor. The patient was supposed to start chemotherapy but was lost for follow up and is likely to have died secondary to the metastatic PGL.

## DISCUSSION

In this study, we have comprehensively defined the genomic profile of PPGL from a previously unstudied highly consanguineous Arab population. Overall, we found a high rate (36.6%) of germline mutations in this series of patients with PPGL without family history of these tumors. In our study, *SDHB* mutations are the most common mutations in PPGL (20.8%) in general and in PGL in particular (30.4%). *SDHC* and *SDHD* mutations are much less common occurring in 3.9% and 3% respectively. Other genetic mutations are rare (Figure 1). None of the more recently described genes (*DLST*, *SLC25A11* and *DNMT3A*) was found mutated in this study. Metastatic PPGL occurred in about 28.6% of patients (6/21) with *SDHB* mutations and in a patient with *SDHAF2* mutation.

Mutations involved in the pathogenesis of PPGL have been recently classified into 4 categories; 1. pseudohypoxemia group involving mainly the succinate dehydrogenase subgroup (*SDHA, SDHB, SDHC, SDHD and SDHAF2*), fumarate hydratase (*FH*) and the *VHL*-*EPAS1* subgroup; 2. The Tyrosine kinase group (*RET, NF1, MAX, TMEM127* and *HRAS*); 3. *WNT*-related pathway (somatic mutations in *MAML3* and *CSDE1*); and 4. adrenocortical admixture group [3]. The last group is less clear than others and its existence is controversial [17, 18].

Our study showed that most mutations involve the pseudohypoxemia group with the vast majority occurring in *SDHB* and to a lesser extent in *SDHC* and *SDHD*. These mutations occurred commonly in PGL but very rarely in PCC. *SDHB* was the most commonly mutated gene (56.8% of patients with positive mutations and 20.8% of all cases studied) and c.268C>T, p.R90X was the most common mutation (Tables 2 and 3). The high frequency of this mutation suggests that it might be a founder mutation in the studied population. By contrast, in the recently published TCGA data from an international consortium population, *SDHB* germline mutations occurred only in 9.8% (17/173 cases) and the p.R90X mutation occurred only in 1/17 cases [3]. The most frequent *SDHB* mutation in TCGA data was p.I127S (5/17 cases), which was not found in our patients [3]. This suggests significant ethnic differences in the molecular genetics of PPGL. By contrast, in the original report of germline mutations in non-syndromic PCC from Europe, *SDHB* mutations were found in 12 of 271 (4.4%) apparently sporadic PPGL (mostly PCC) [2] which is not much different from our study in which 2 out of 32 (6.3%) PCC carry *SDHB* mutations. A more recent report from Spain in which 329 sporadic single non-familial PPGL were tested for mutations showed an overall prevalence of germline mutations of 14%. Similar to our study, PGL were more commonly mutated (28.7% vs. 42% in our study) and most mutations occurred in *SDHB* (63% vs. 56.8% in our study) followed by *SDHD* (13% vs. 8% in our study) and *SDHC* (4.3% vs. 10.8% in our study) with other genes being rarely mutated [19]. In another study from USA, a review of 129 cases of PPGL who underwent surgery was undertaken and of 42 patients that were tested, 21 (50%) were positive for germline mutations. However, some of those cases were syndromic PPGL [20]. Another study from India reviewed 150 cases of PPGL of whom 30 cases were syndromic PPGL. These cases were tested only for 5 genes (*RET, VHL, SDHB, SDHD and SDHC*). All 30 syndromic PPGL were positive for germline mutations while in 120 cases of this series with sporadic PPGL, 19 (15.8%) had germline mutations with *VHL, SDHB* and *SDHD* being the most commonly mutated genes [21].

In the current study, *SDHC* mutations were the second most common mutations in PGL occurring in 4 out of 37 cases (10.8%) with positive mutations. This is unusual as the literature cited *SDHC*-associated PGL to be much less common than *SDHB* and *SDHD*-associated PGL [22–25]. In a review of a number of series with more than 3000 patients with PPGL, *SDHC* mutations occurred only in 1% of cases (31/3193 cases) [1]. In the TCGA data, *SDHC* mutations were not found [3]. Similarly, in a recent report from Europe, *SDHC* mutations were not found in 87 cases of PCC [24]. An *SDHC* founder mutation (c.397C>T, p. Arg133Ter) was detected in about 70% of a cohort of 29 French Canadian patients presenting mostly (70%) with head and neck PGL and with distant metastasis in 30% of them [26]. In our study, two of the *SDHC*-associated PGL occurred in the head and neck and two were abdominal PGL. All of these 4 cases were benign PGL. Other studies have shown that *SDHC*-related PGL commonly occur in the head and neck region but also in the mediastinum and abdominal regions [22, 23].

*SDHD* mutations occurred in three cases with head and neck PGL, two unilateral and one bilateral. Apart from these 3 cases, the most common *SDHD* variant found in this study was the c.34G>A, p.G12S occurring in 7 cases. The pathogenicity of this variant has been controversial with some studies suggesting that it is pathogenic while others suggested that is a non-pathogenic SNP [27–29]. This variant was not reported in the TCGA data [3]. If considered pathogenic, *SDHD* mutations would be the second most common mutations after *SDHB* (10/48, 20.8%). However, we considered it to be non-pathogenic as it also occurred in the normal Saudi Genome Project database (MIF 0.046%). In fact, all *SDHD* mutations were also rare in the TCGA data occurring only in 3 out of 173 cases (2%) [3]. However, TCGA study excluded head and neck PGL in which *SDHD* mutation most commonly occur [30].

Other pseudohypoxia genes were much less frequently mutated. *SDHA* mutations occurred only once in a patient with carotid body tumor (Table 4). Mutations in this gene are rare but can be associated with aggressive metastatic PGL similar to those of *SDHB* [31, 32]. One patient had an *SDHAF2* variant of unknown significance and presented with metastatic PCC. *SDHAF2* mutations are extremely rare and their association with metastatic PPGL has not been reported. However, this variant was assessed to be disease causing by mutation taster and probably damaging with a score of 0.999 on Polyphen2 analysis. In a large study from Spain and Netherland with more than 430 patients with *SDHB*- and *SDHD*- negative sporadic and familial PPGL tested specifically for *SDHAF2*, none was found to have *SDHAF2* mutation [33]. *SDHAF2* mutations were not also reported in TCGA data [3]. *VHL* mutations were detected in two patients; one with unilateral and one with bilateral PCC. *VHL* mutations have been commonly reported in hereditary PPGL and can be associated with unilateral or bilateral PCC or less frequently with PGL [2, 34].

Of the tyrosine kinase group, *RET* mutation occurred in only 1 case of abdominal PGL. Interestingly, *MAX* mutations occurred in 2 cases of PCC, both of whom presented with bilateral PCC. *MAX* mutations-associated PCC tend to be bilateral and may metastasize to distant sites [35]. In a study of 972 cases from the European-American-Asian Pheochromocytoma and Paraganglioma Registry without mutations in the common PPGL genes, 58 had mutations in less commonly mutated genes (*SDHA, TMEM127, SDHAF2*) including 8 cases of PCC with *MAX* mutations. Two of these 8 cases were bilateral PCC [32]. We also found one case with a *TMEM127* mutation in an abdominal PGL. *TMEM127* mutations are generally more frequent than *MAX* mutations and may present with PCC, abdominal or head and neck PGL [32, 36].

Distant metastases are associated with unfavorable prognosis and are essentially incurable [37, 38]. Genetic markers that may predict development of metastatic PPGL can be of significant value for early and more effective intervention and closer follow up [39–44]. *SDHB* mutations have been associated with increased risk of distant metastasis [45–49]. The association between other gene mutations and distant metastasis are less clear [40, 43]. In this study, *SDHB* mutations remain the most frequently associated mutations with distant metastasis occurring in 6 out of 21 cases (28.6%). Only one additional case with an *SDHAF2* variant developed distant metastasis. However, 3 cases with distant metastasis had no detectable mutations in any of known or potential candidate gene.

Our study is the first to evaluate the rates, types of germline mutations and the phenotype-genotype correlation in a large series of PPGL from an Arab population. However, it has some shortcomings including limitation of our study to germline mutations. Some previously reported mutations are somatic, particularly the *WNT*-related *MAML4* fusion and *HRAS* mutations [3]. We have not included patients with positive family history of PPGL since our aim was to assess the rates of undiagnosed hereditary cases and to discover cases that seem sporadic. However, data from this study from an Arab population strongly supports genetic screening for patients with PPGL in this population since the frequency of germline mutations is high. It also suggests that for targeted sequencing, *SDHB* should be the first gene to be tested, especially in patients with abdominal or metastatic PGL. Other genes, especially *SDHC* and *SDHD* should be tested when mutations are not found in *SDHB* or the clinical presentation suggests a likely genetic mutation (e. g. *VHL* and *MAX* in bilateral PCC).

In conclusion, approximately 37% of our patients with non-familial PPGL harbor germline mutations in different susceptibility genes. The most commonly mutated gene is *SDHB* presenting mostly with abdominal PGL and less frequently with head and neck PGL. It is also associated with the highest risk of distant metastasis. *SDHC* and *SDHD* mutations are the second most common genetic alterations in PGL. *VHL* and *MAX* mutations occur mainly in PCC and tend to present with bilateral disease. Other genetic alterations are rare but have unique presentations.

## PATIENTS AND METHODS

### Patients

We studied all patients of PPGL who have no definite family history of such tumors and were seen at the King Faisal Specialist Hospital and Research Center (KFSHRC), Riyadh, Saudi Arabia during the period of January 2003-January 2019. KFSHRC is the main tertiary care center in Saudi Arabia where most cases of PPGL are referred to. Over this period, we managed 101 patients with non-familial PPGL. To avoid selection bias, we excluded patients with known familial PPGL syndromes. All cases underwent surgery and the histopathological examination confirmed the diagnosis of PPGL. The patients’ characteristics are summarized in Table 1.

### Samples

We obtained an Institutional Review Board approval from the Office of Research Affairs of the KFSHRC. Informed consents for all prospective blood sample collection were obtained from the patients or their guardians. We collected blood samples from 53 cases. However, in cases where blood samples were not available for testing, we used formalin fixed paraffin embedded (FFPE) non-tumorous tissue (48 patients). To ensure that testing is for germline mutations, the tissue was carefully selected by an experienced pathologist (H. A) from previously surgically removed normal tissues avoiding any tumor tissue. Genomic DNA was isolated from peripheral blood leucocytes using the Gentra Blood Kit (Qiagen Corp, Valencia, CA, USA) according to the manufacturer’s instructions. For isolation of DNA from FFPE, DNA was extracted using a commercial DNA extraction kit (QIAamp DNA FFPE Tissue Kit, QIAGEN, Catalog No. 56404) according to the manufacturer’s instructions. DNA was quantified using a nanodrop2000 spectrophotometer (Thermo Scientific, Wilmington, DE, USA) and its purity was assured by the A260/280 ratio of ≥ 1.8 indicating good purity DNA. We performed polymerase chain reaction (PCR) and direct Sanger sequencing using Big Dye terminator v3.1 cycle sequencing reaction kit and an ABI PRISM 3730XL genetic analyzer (Applied Biosystems) to detect mutations in *RET, SDHA, SDHB, SDHC, SDHD, SDHAF2, VHL, MAX* and *TMEM127*. The primers and PCR conditions have been published previously [2, 11, 50]. When a pathogenic mutation was found in one of these genes, no further testing was performed in the remaining genes. This approach led to identification of 30 pathogenic or likely pathogenic variants in any one of these genes (Table 2). We subjected the remaining cases in whom no mutation was found by PCR and Sanger sequencing or when Sanger sequencing was unsuccessful or unclear to NGS-based whole exome analysis. Variants reported by NGS were subsequently confirmed by direct Sanger Sequencing.

### Whole exome sequencing

Whole exome sequencing was achieved using the Ion Proton platform (AmpliSeq kit). Briefly, 100 ng DNA from each sample were collected and the extracted DNA is then amplified using AmpliSeq HiFi mix (Life Technologies, Carlsbad, CA, USA) for 10 cycles. The resultant PCR products were then pooled followed by primer digestion using FuPa reagent (Life Technologies, Carlsbad, CA, USA). A ligation step was then conducted using Ion P1 and Ion Xpress Barcode adapters. After that the libraries were purified and quantified using qPCR and the Ion Library Quantification Kit (Life Technologies, Carlsbad, CA, USA). The next step included emulsion of the libraries using Ion OneTouch System to attach the DNA fragments to the Ion Sphere particles. The final step in the library preparation included enrichment of the Ion Sphere particles using Ion OneTouch ES (Life Technologies, Carlsbad, CA, USA). Once the library became ready, they are loaded on the sequencing chip which is then inserted into the Ion Proton instrument (Life Technologies, Carlsbad, CA, USA) for sequencing.

### Bioinformatics analysis

For bioinformatics analysis, we used the Torrent Suite (https://github.com/iontorrent/TS) analysis kit, using the manufacturer’s recommended parameters for base calling and alignment. The first step after base calling is to check the reads for quality and trim the low-quality parts. Then the reads are aligned to the reference human genome (version hg19) using the manufacturer’s recommended parameters. After the alignment, we used the variant calling pipeline of the Torrent suite both of which are based on the BWA-GATK pipeline, but they were more tuned to the Ion Torrent technology, by including flow signal information and library of common sequencing error motifs to improve the accuracy.

After variant calling, we ran the in-house developed annotation pipeline. This pipeline is based on the Annovar package (http://annovar.openbioinformatics.org), which includes about 40 databases. We also added more information tracks including variant frequencies from the database of the Saudi Human Genome Program.

To speed up the analysis, the list of annotated variants per each sample was filtered to remove intronic and synonymous variants. The remaining variants were then prioritized based on the following criteria: 1) Existence in the set of genes which are known to be related to the disease, 2) the effect score (whether the variant is truncating/damaging or not), 3) frequency in public databases and Saudi population.

### Quality of sequencing

The sequencing quality was assessed using different metrics. These showed that the target regions are well covered by the NGS reads (99.6% total average coverage at 1× and 96.88% average coverage at 20×) with an average depth of 224 (i. e. each base in the target region is covered by 224 reads on average). Genetic variants detected by NGS were subsequently confirmed by PCR and targeted Sanger sequencing of the exons/introns in which these variants were found.
